# Serotonergic neurons respond to nutrients and regulate the timing of steroid hormone biosynthesis in *Drosophila*

**DOI:** 10.1038/ncomms6778

**Published:** 2014-12-15

**Authors:** Yuko Shimada-Niwa, Ryusuke Niwa

**Affiliations:** 1 Faculty of Life and Environmental Sciences, University of Tsukuba, Tennoudai 1-1-1, Tsukuba 305-8572, Japan; 2 PRESTO, JST, 4-1-8 Honcho, Kawaguchi 332-0012, Japan

## Abstract

The temporal transition of development is flexibly coordinated in the context of the nutrient environment, and this coordination is essential for organisms to increase their survival fitness and reproductive success. Steroid hormone, a key player of the juvenile-to-adult transition, is biosynthesized in a nutrient-dependent manner; however, the underlying genetic mechanism remains unclear. Here we report that the biosynthesis of insect steroid hormone, ecdysteroid, is regulated by a subset of serotonergic neurons in *Drosophila melanogaster*. These neurons directly innervate the prothoracic gland (PG), an ecdysteroid-producing organ and share tracts with the stomatogastric nervous system. Interestingly, the projecting neurites morphologically respond to nutrient conditions. Moreover, reduced activity of the PG-innervating neurons or of serotonin signalling in the PG strongly correlates with a delayed developmental transition. Our results suggest that serotonergic neurons form a link between the external environment and the internal endocrine system by adaptively tuning the timing of steroid hormone biosynthesis.

## Abstract (web summary)

Steroidal hormones play a major role in the transition from juvenile-to-adult stages of development. Here, Shimada-Niwa and Niwa show that production of one such hormone in the prothoracic gland of *Drosophila melanogaster*, is regulated by a subset of serotonergic neurons innervating the prothoracic gland.

## Graphical abstract

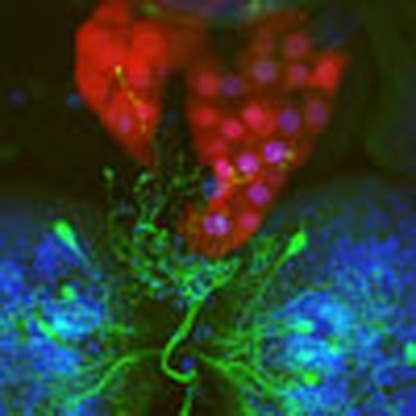

Steroid hormones play crucial roles in many aspects of development, growth and reproduction. They have a conserved role in controlling the developmental transition from juvenile-to-adult across animal phyla. For example, human steroid hormones promote the development of secondary sexual characteristics at puberty, leading to adult sexual maturation^1^. The insect steroid hormone ecdysteroid determines the timing of moulting and metamorphosis^2^. Interestingly, the temporal coordination of steroid hormone biosynthesis during the juvenile-to-adult transition is tightly coupled to the nutrient conditions in the juvenile stage, which allows organisms to increase their survival fitness and reproductive success^3^. However, it remains unclear how nutrient information is incorporated to control the timing of steroid hormone biosynthesis.

The fruit fly *Drosophila melanogaster* provides a suitable model for studying the regulatory system of steroid hormone/ecdysteroid biosynthesis^4^,^5^. During the larval stages, a form of ecdysteroid, ecdysone (E), is synthesized in a special endocrine organ called the prothoracic gland (PG; Fig. 1a,b). Studies during the past decade have successfully identified ecdysteroidogenic enzyme genes acting in the PG, such as *neverland* (*nvd*), *shroud* (*sro*), *spookier* (*spok*), *phantom* (*phm*), *disembodied* (*dib*) and *shadow* (*sad*), which mediate the steps converting cholesterol to E (ref. 6). Once released into the haemolymph, E is further converted to an active form of ecdysteroid, 20-hydroxyecdysone (20E), in peripheral tissues by the action of *shade*^6^. The level of ecdysteroids (E and 20E) is increased and decreased in a stage-specific manner, controlling a battery of downstream gene expression profiles^7^.

The biosynthesis of E and 20E is controlled in response to several environmental parameters including nutrition, temperature and light^2^,^3^. The environmental information is transduced in the PG through neuronal inputs or humoral factors. A well-known example is prothoracicotropic hormone (PTTH)-producing neurons, which directly innervate the PG and control E biosynthesis via Torso–ERK signalling^8^,^9^,^10^. When PTTH neurons are genetically ablated or Torso–ERK signalling is impaired in the PG, the timing of ecdysteroid biosynthesis is delayed in the larva-to-pupa transition (pupariation). As a result, these animals extend the duration of larval growth, giving rise to giant-size larvae and pupae^8^,^9^. Because PTTH neurons are connected to clock neurons^8^,^11^, PTTH signalling is hypothesized to respond to light^10^. Furthermore, *ptth* expression patterns are affected by an impairment of imaginal disc growth^12^,^13^,^14^. Another example is *Drosophila* insulin-like peptides, which are produced in and secreted from the insulin producing cells in a nutrient-dependent manner. Several lines of evidence indicate that the insulin/insulin-like growth factor-1 signalling (IIS) pathway and the target of rapamycin (TOR) pathway in the PG control the duration of larval growth^15^,^16^,^17^,^18^. The expression levels of both *torso* and *insulin receptor (inr)* are regulated by the transforming growth factor-β (TGF-β) signalling pathway^19^, indicating that multiple signalling pathways are coordinated for a convergence of signalling output: a time for ecdysteroid biosynthesis in the juvenile-to-adult transition.

Although PTTH neurons are the only neurons so far known to directly innervate the PG^20^, other neurons are known to project to the PG in lepidopteran species^21^, implying that uncharacterized neurons projecting to the PG also exist in *D. melanogaster*^5^. Here, we identified a subset of serotonin (5-hydroxytryptamine, 5-HT)-producing neurons that directly innervate the PG. These neurons belong to the stomatogastric nervous system (SNS) and respond to nutrient conditions. Our results suggest that three pairs of serotonergic neurons are responsible for pupariation timing and that serotonin signalling is a novel regulator for ecdysteroid biosynthesis. Based on these results, we propose that serotonergic neuronal control mediates the nutrient-dependent developmental plasticity via modulating steroid hormone biosynthesis in the juvenile-to-adult transition.

## Results

### A subset of serotonergic neurons directly innervates the PG

**Figure 1:**
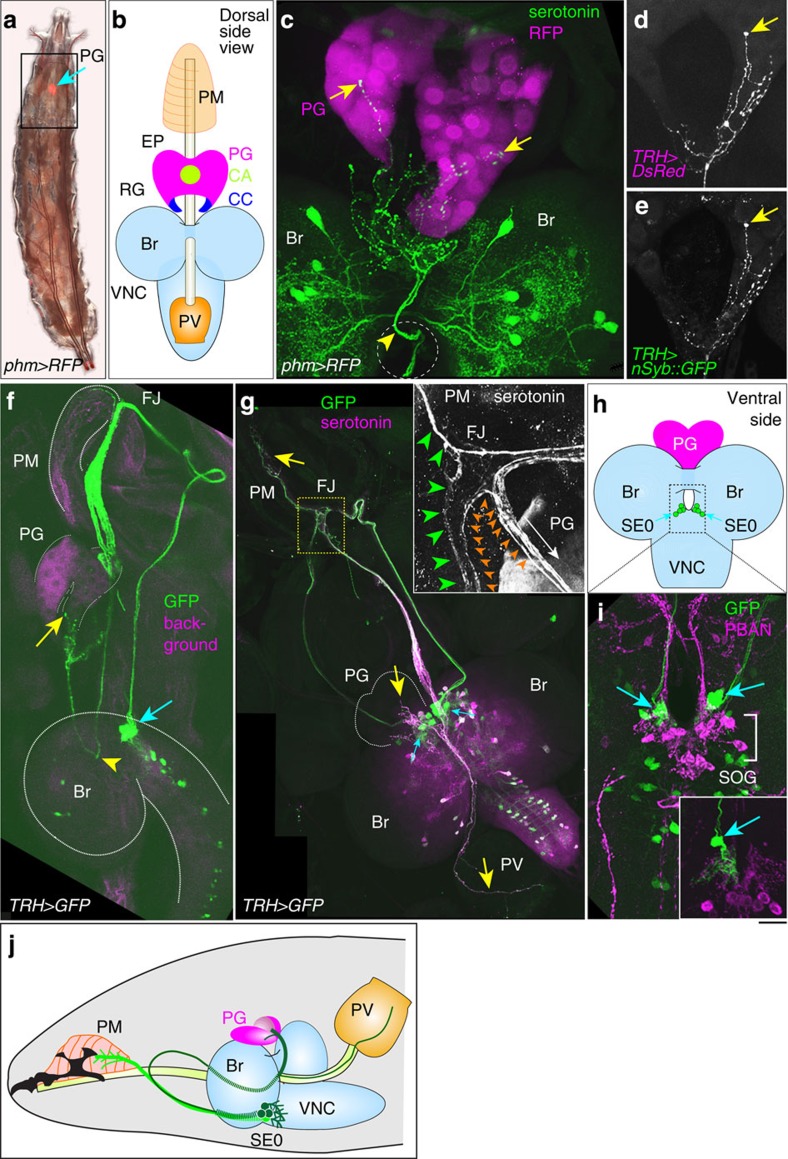
Serotonergic SE0_PG_ neurons innervate the PG. (**a**) The third instar larva expressing *RFP* using *phantom–GAL4* (*phm>RFP*). The anterior side is at the top. *RFP* is expressed in the prothoracic gland (PG, arrow). The boxed area is illustrated in **b**. (**b**) The pharyngeal muscles (PM), oesophagus (EP), ring gland (RG), brain (Br), ventral nerve cord (VNC) and proventriculus (PV). The RG contains the PG, the corpora allata (CA) and the corpora cardiaca (CC). (**c**) The Br–RG complex from a *phm>RFP* third instar larva was immunostained for serotonin (green). Serotonergic neurons directly innervate the PG (arrows). The neurites pass through the oesophagus foramen (arrowhead, outlined circle). (**d**,**e**) The PG-projecting neurons were visualized with DsRed and nSyb::GFP using *TRH–GAL4*. (**f**) A *TRH>GFP* third instar larva was dissected from the lateral side. PG-projecting neurons (green, yellow arrow) passed through the oesophagus foramen (arrowhead, see also **c**), extending towards the frontal nerve junction (FJ). The blue arrow indicates the ‘SE0’ cluster in the ventral side of the brain. Magenta is used as a background colour to show the shapes of the tissues. (**g**) A *TRH>GFP* third instar larva was dissected from the dorsal side and immunostained for serotonin (magenta) and GFP (green). The SE0 neurons (blue arrows) innervated the PG as well as the PM and the PV (yellow arrows). The boxed area is magnified in the inset. At the FJ, the neural tracts bifurcated to PM and PG (green and orange). (**h**) Four pairs of SE0 cells (circles). The boxed area is shown in **i**. (**i**) The *TRH>GFP* third instar larva was immunostained for GFP (green) and a suboesophageal ganglion (SOG) marker PBAN (magenta). The SE0 neurons (arrows) are located anterior to the SOG cells (bracket). The inset is a single-cell clone of SE0 neurons. (**j**) The anterior half of a larva and the tracts of SE0 neurons (green lines) are illustrated. The scale bar depicted in **i** corresponds to 481 μm (**a**), 18.7 μm (**c**), 20.0 μm (**d**,**e**), 32.7 μm (**f**), 50 μm (**g**), 28.4 μm (**g**, inset), 28.1 μm (**i**) and 24.4 μm (**i**, inset).

In the course of studying how neural inputs affect E biosynthesis in the PG, we focused on a subset of serotonergic neurons that innervate the ring gland (RG). The RG is a composite endocrine organ in cyclorrhaphous Diptera, including *D. melanogaster*, consisting of the PG, the corpora allata and the corpora cardiaca (CC; ref. 22). Previous studies report that serotonergic neurons innervate the CC area of the RG (refs 20, 23, 24). Using the *phantom (phm)–GAL4#22* driver, which is specifically expressed in the PG cells, we found that serotonin-immunoreactive neurites directly innervated the PG cells as well as the CC (Fig. 1c, arrows). These neurites were also labelled with *tryptophan hydroxylase (TRH)–GAL4*, which is highly selective for serotonergic neurons (Fig. 1d–i)^25^. n-Synaptobrevin (nSyb)::green fluorescent protein (GFP) was localized to the neurite termini, indicating that they are the presynaptic sites (Fig. 1e). Next, we looked for cell bodies of the PG-innervating serotonergic neurons by tracing backwards through the axons. Notably, the axons pass through the oesophagus foramen between the brain hemispheres (Fig. 1c,f, arrowheads) and extend towards the pharyngeal region (Fig. 1f,g). At the frontal nerve junction (an inset in Fig. 1g), the axons turn back to the brain (Fig. 1f,g) and reach the cell bodies at the tritocerebral compartment on the ventral side (Fig. 1f–i, blue arrows). The location of the cell bodies seems to correspond to the ‘SE0’ cluster, where their dendrites extend to the region of the suboesophageal ganglion (SOG, Fig. 1i)^24^,^26^. It should be noted that the cell bodies of SE0 neurons were faintly immunostained with anti-serotonin antibody (Fig. 1g), which would explain why these neurons have not been defined in the classic anatomical studies^20^,^23^. We hypothesize that SE0 neurons extend so far that serotonin is mostly transported to the terminal region and that little remains localized in the cell bodies. In our observation, four pairs of ‘SE0’ neurons project not only to the PG but also to the feeding apparatuses, such as the pharyngeal muscles (PM) and proventriculus (insect foregut), suggesting that the ‘SE0’ cluster neurons belong to the stomatogastric (enteric) nervous system (Fig. 1g,j)^24^,^26^. To discriminate the serotonergic neurons innervating the PG from other serotonergic neurons in the ‘SE0’ cluster, we denote the serotonergic neurons innervating the PG as ‘SE0_PG_ neurons’.

### Three pairs of serotonergic neurons innervate the PG

**Figure 2:**
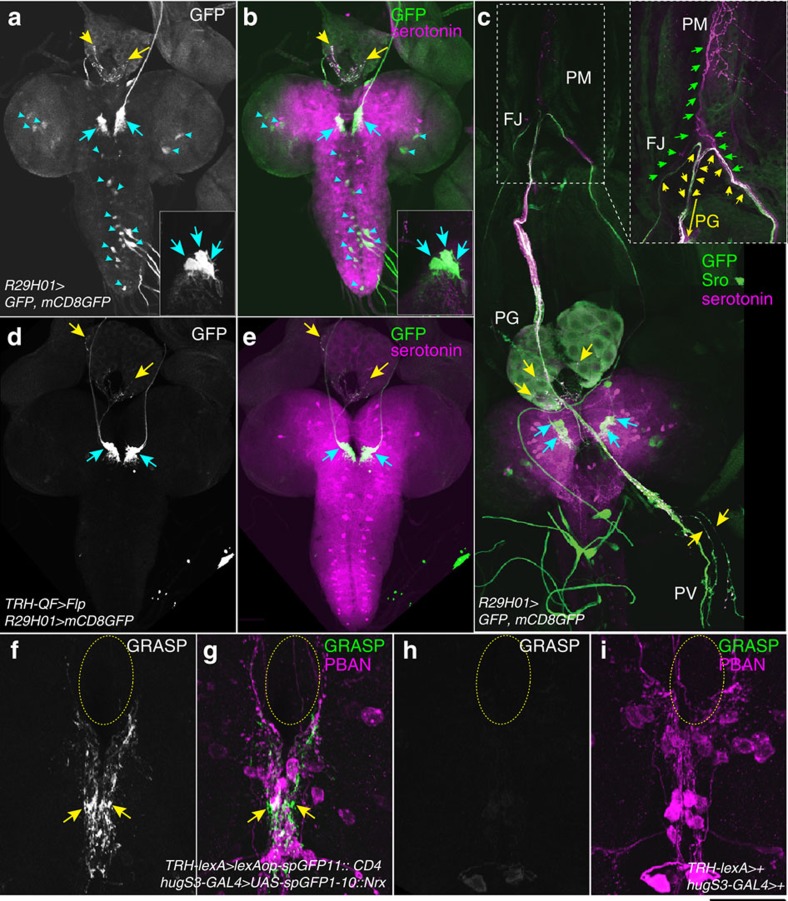
*TRH–GAL4* and *R29H01–GAL4* share three pairs of neurons in the SE0 cluster. (**a**,**b**) The Br–RG complex from the late third instar larva expressing *R29H01>GFP* was immunostained with anti–GFP (**a** and green in **b**) and anti-serotonin antibodies (magenta in **b**). GFP was detected in the PG-projecting neurites (yellow arrows), three pairs of SE0 cluster cells (blue arrows, insets), and other cells that were not serotonin immunoreactive (blue arrowheads). (**c**) The dorsal side of the third instar larva expressing *R29H01>GFP*. The PG and serotonergic neurons were visualized with anti-Shroud (Sro, green) and anti-serotonin (magenta) antibodies, respectively. The boxed area is magnified in the inset. *R29H01–GAL4*-driven *GFP* expression was detected in the PG or PV-projecting neurons (yellow arrows), but not in the PM-projecting neurons (green arrows in the inset). At the FJ, the neural tracts bifurcated to PM and PG (green and yellow arrows). (**d**,**e**) Only three pairs of SE0 neurons (blue arrows) are labelled with GFP (**d** and green in **e**) as well as serotonin (magenta in **e**). Any of these cells innervated the PG (yellow arrows). By using the Q system in conjunction with the GAL4 system, *R29H01–GAL4*-driven *GFP* was expressed only in the overlapped region where both *TRH–QF* and *GAL4* are expressed. (**f**–**i**) In GRASP analysis, *spGFP1-10::Nrx* was expressed in SOG neurons with *hugS3–GAL4*, and *spGFP11::CD4* was expressed in serotonergic neurons with *TRH–lexA*. GRASP signal was detected between SOG and SE0 neurons with anti–GFP body (green, arrows). In contrast, negative controls did not give signals (**h**,**i**). SOG neurons were labelled with anti-PBAN antibody (magenta). The oesophagus foramen is outlined with yellow-dotted lines. The scale bar depicted in **i** corresponds to 183.8 μm (**a**,**b**,**d**,**e**), 126.3 μm (**c**), 101.8 μm (**c**, inset) and 50.0 μm (**f**–**i**).

Because *TRH–GAL4* is expressed in almost all serotonergic neurons^25^, we searched for an alternative *GAL4* driver that is expressed in a smaller subset of neurons including SE0_PG_ neurons. In the *GAL4* collection of the Janelia FlyLight database^27^, we found that *R29H01–GAL4*, which contains a genomic fragment of gene *CG8742* (*Gyc76C*), was expressed in SE0_PG_ neurons (yellow arrows, Fig. 2a–c) and some other non-serotonergic neurons (Fig. 2a,b, blue arrowheads). In the SE0 cluster, the *GAL4*-driven *GFP* expression was detected in three pairs of cells (insets in Fig. 2a,b), indicating that *R29H01–GAL4* refines serotonergic, *TRH–GAL4*-positive neurons innervating the PG. Among these three pairs of neurons, we failed to narrow down how many neurons innervate the PG with single-cell clone analysis. Because *R29H01–GAL4*-driven *GFP* was not expressed in the neurites projecting to the PM (an inset in Fig. 2c), the PM is specifically innervated by at least one pair of the SE0 cluster neuron that is *R29H01–GAL4* negative and *TRH–GAL4* positive (Fig. 1j).

To confirm that *R29H01–GAL4*-positive neurons other than SE0_PG_ neurons are non-serotonergic, we used the Q system in conjunction with the GAL4 system to generate intersectional expression patterns^28^. In our crossing scheme, *TRH–QF* induces *QUAS–FLP* expression, which removes the transcription stop cassette, allowing for *R29H01–GAL4*-induced *GFP* expression only in the overlapped region where both *QF* and *GAL4* are expressed. We found that *GFP* expression was limited to three pairs of cells in the SE0 cluster (Fig. 2d,e). This observation indicates that the expression patterns of *R29H01–GAL4* and *TRH–GAL4* share only three pairs of SE0 neurons.

The SE0 cluster neurons are located just anterior to the SOG (Fig. 1i), implying that SE0 neurons may have synaptic contacts with SOG neurons. The SOG is proposed to act as a feeding control centre in insects^29^, expressing a neuropeptide called Hugin^30^,^31^. To test this idea, we employed an enhanced variant of GFP reconstitution across synaptic partners (GRASP)^32^ in which *UAS–spGFP1-10::Neurexin* is expressed under the control of *hugS3–GAL4*, and *LexAop–spGFP11::CD4* is expressed under the control of *TRH–LexA*^33^. SpGFP1-10::Neurexin is targeted to synapses of SOG neurons, whereas spGFP11::CD4 permits cell-surface expression on SE0 neurons. Reconstituted GFP signals were detected in the region between SOG and the SE0 cluster (Fig. 2f,g), whereas negative controls did not give GFP signals (Fig. 2h,i). This result supports the idea that SE0 neurons receive some signals from SOG neurons.

### The projection of SE0_PG_ neurons responds to nutrient

**Figure 3:**
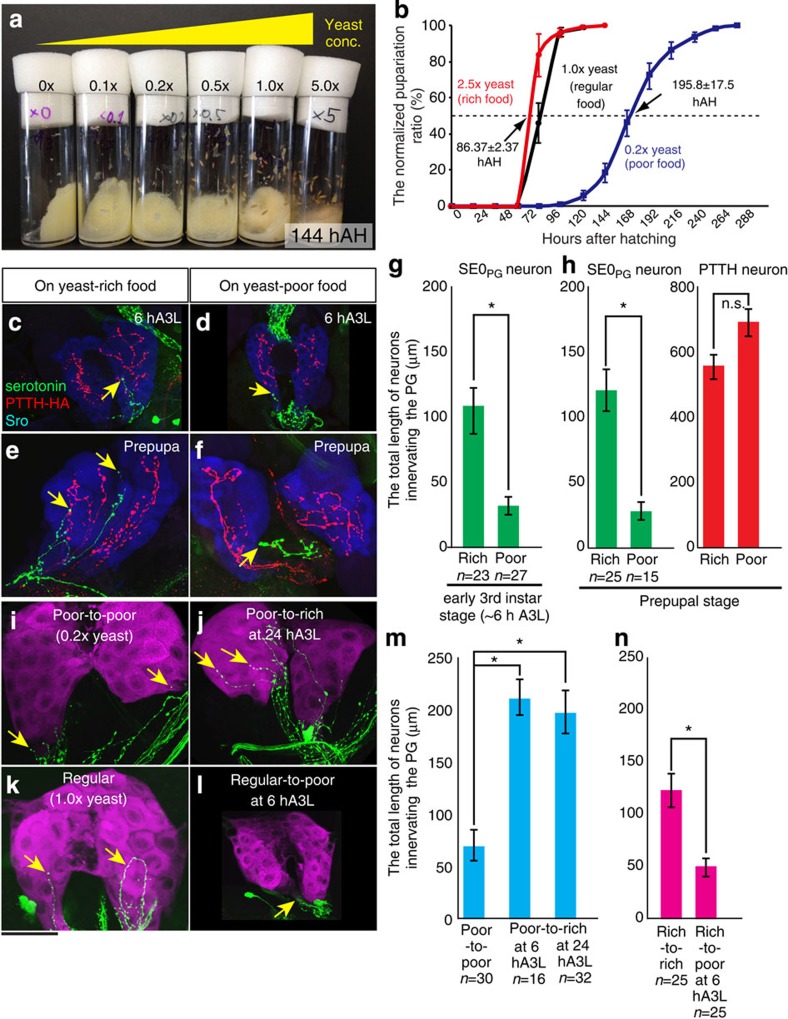
The projection of SE0_PG_ neurons is affected by nutrient conditions. (**a**) The pupariation timing depends on the yeast concentration in food. The photo was taken at 144 h after hatching (hAH). While almost all animals pupariate on food containing 1.0–5.0 × yeast (2.0–10.0 g of yeast per 50 ml), larvae still remain in food containing 0–0.5 × yeast. Fifty larvae were placed in a 20 ml vial. (**b**) The normalized pupariation timing. In this and the subsequent figures, the percentages were normalized to the final number of pupae. Error bars represent s.e.m. Under the regular food condition, larvae pupariate at 96–120 hAH at 25 °C (*n*=274, 1.0 × yeast, black line). In contrast, 50% of larvae became pupae at 86.37±2.37 hAH under the rich food condition (*n*=324, 2.5 × yeast, red line) and at 195.8±17.5 hAH under the poor food condition (*n*=214, 0.2 × yeast, blue line). (**c**–**f**) The projections of SE0_PG_ neurons (green) and PTTH neurons (red) were visualized with anti-serotonin and anti–HA antibodies in larvae expressing genomic *ptth–HA*. The PG was labelled with Sro (blue). The projecting neurites are indicated by arrows in the early third instar stage (0–6 h after L2–L3 moulting, A3L, **c**,**d**) and the prepupal stage (**e**,**f**). (**g**,**h**) The total length of SE0_PG_ and PTTH neurons innervating the PG was measured in the early third instar and the prepupal stage. (**i**–**n**) SE0_PG_ neurons respond promptly to food signals. When larvae were transferred from yeast-poor to yeast-rich food in the third instar stage, the SE0_PG_ projections recovered after 2 days (**i**,**j**). The total length of the SE0_PG_ projections was increased (**m**). Conversely, when larvae were transferred from regular food to yeast-free food in the early third instar stage, the SE0_PG_ projections were severely disrupted (**k**,**l**). Note that the PG became smaller in **l**. The total length of projecting neurites was decreased (**n**). For statistical analysis in **g**,**h**,**m**,**n**, the mean values of 15–32 samples are shown with s.e.m. in each condition. Student’s *t*-test, *P<0.01. Bar, 50 μm. Each experiment was conducted independently at least four times.

The prominent anatomical features of SE0_PG_ neurons described above prompt us to think that the innervation of SE0_PG_ neurons is related to nutrient signals or feeding behaviours. Indeed, the timing of ecdysteroid biosynthesis depends on nutrient conditions^16^, making SE0_PG_ neurons candidates for transmitting nutrient signals to the PG. To test our hypothesis that the serotonergic neurons respond to nutrient conditions, we examined the morphology of their neurites under various food conditions. When the first instar larvae were raised on standard agar–cornmeal food containing varying amounts of yeast, the timing of pupariation varied (Fig. 3a). On our regular food condition (1.0 × yeast=2.0 g of yeast per 50 ml), almost all larvae became pupae in 96–120 hours after hatching (hAH) at 25 °C (black line in Fig. 3b). In contrast, the timing of pupariation was delayed by 4–5 days under the yeast-poor condition (0.2 × yeast, blue line in Fig. 3b) compared with the yeast-rich condition (2.5 × yeast, red line in Fig. 3b). In the yeast-rich condition, SE0_PG_ neurons projected well to the PG at the early third instar stage (0–6 hours after L2–L3 moulting (hA3L); Fig. 3c), as well as the prepupal stage (Fig. 3e). In contrast, SE0_PG_ neurons barely projected to the PG under the yeast-poor condition (Fig. 3d,f). Both serotonin signals and the membrane-associated mCD8::GFP signals in the neurites were affected (Supplementary Fig. 1a–f), indicating that the axon terminal morphology changed under the yeast-poor condition. The total length of SE0_PG_ neurons innervating the PG was significantly decreased under the yeast-poor condition (Fig. 3g,h). This difference may be specific to SE0_PG_ neurons because the projection of PTTH neurons was not affected under the yeast-poor condition (Fig. 3c–f and h). We would like to emphasize that we carefully dissected the larvae (see the Methods section) and observed both SE0_PG_ and PTTH neurons simultaneously in the same, not different, samples. Therefore, it is unlikely that the differences in the neurite projection length were due to an artifact of dissection. Taken together, these results support the idea that SE0_PG_ neurons respond to food-related signals.

To examine the plasticity of the neurites of SE0_PG_ neurons, we switched the food condition from yeast poor to yeast rich and *vice versa* during the third instar larval stage. We confirmed that SE0_PG_ neurons innervated the PG in the first instar stage before food uptake (Supplementary Fig. 1g–i). When the first instar larvae were raised on yeast-poor food and then transferred to yeast-rich food in the third instar stage, the projection of SE0_PG_ neurons recovered 2 days after the transfer (Fig. 3i–m). Correspondingly, the delayed timing of pupariation was also recovered (Supplementary Fig. 1j). Conversely, when larvae were transferred from regular food (1 × yeast) to yeast-free food (0 × yeast) in the early third instar stage (0–6 hA3L), SE0_PG_ neurons hardly projected to the PG (Fig. 3k–n). In this case, the timing of pupariation was delayed, and the PG cells decreased in size (Fig. 3l and Supplementary Fig. 1j). These results suggest that SE0_PG_ neurons respond reversibly to nutrient signals and adjust the timing of pupariation to the nutritional conditions.

### SE0_PG_ neurons modulate the timing of E biosynthesis

**Figure 4:**
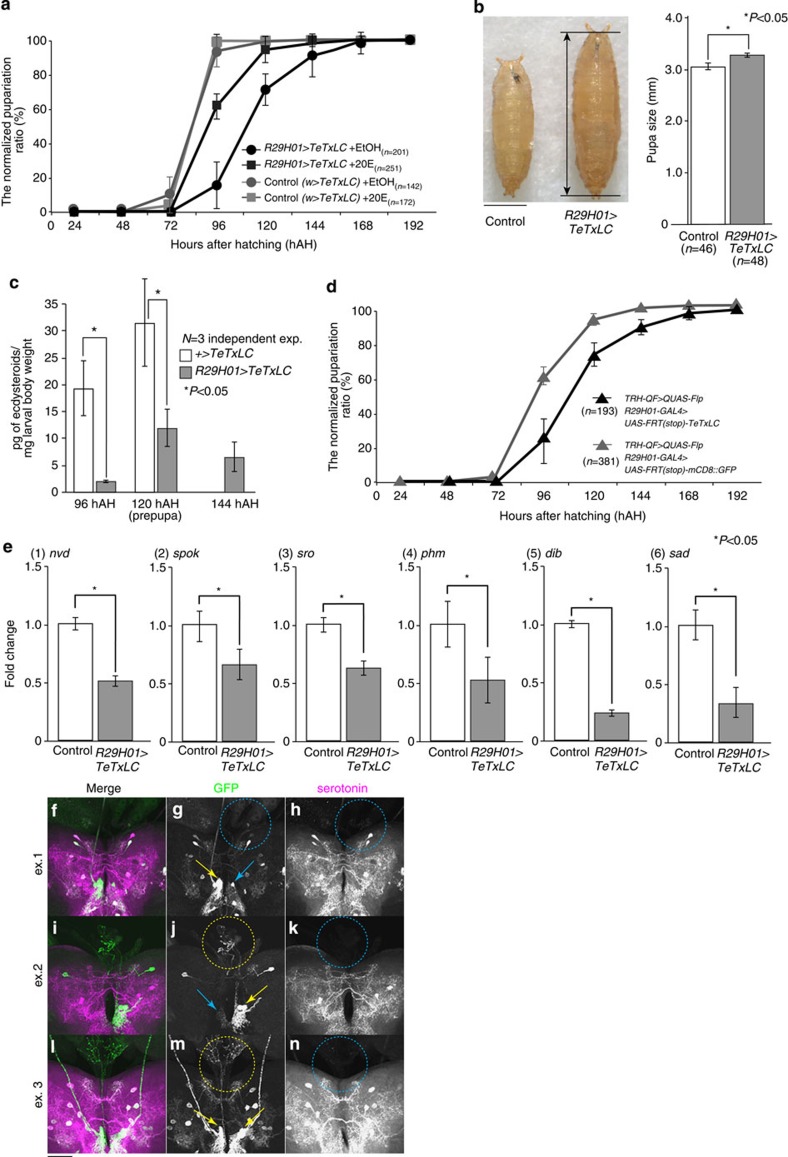
Inhibition of SE0_PG_ neurons is coupled to delayed pupariation. (**a**) The pupariation ratio after hatching. When newly hatched larvae were raised at 29 °C, control larvae pupariate at 72–96 hAH. In contrast, *TeTxLC*-expressing larvae delayed pupariation by 1–2 days, and the delay was rescued by feeding with 20E. The number of animals: *R29H01>TeTxLC* (*n*=201, EtOH; *n*=251, 20E), *w>TeTxLC* (*n*=142, EtOH; *n*=172, 20E). (**b**) The prolonged larval growth period resulted in an increased pupal size. The pupal size (double-headed arrow) was compared between control (*n*=46) and *R29H01>TeTxLC* (*n*=48). (**c**) The levels of ecdysteroids in *w>TeTxLC* and *R29H01>TeTxLC* animals at 96–144 hAH. At 120 hAH, prepupae were collected from the control. (**d**) The pupariation timing was delayed after *TeTxLC* was expressed in three pairs of SE0 neurons (black, *n*=193). *mCD8::GFP* was expressed in these neurons of control (grey, *n*=381). (**e**) The expression of ecdysteroidogenic genes decreased in the RG of *R29H01>TeTxLC* larvae at 96 hAH. The fold changes compared with control larvae (*w>TeTxLC*) were calculated. (1) *nvd*, (2) *spok*, (3) *sro*, (4) *phm*, (5) *dib*, (6) *sad*. (**f**–**n**) Three examples of the third instar Br–RG complex containing MARCM clones that express *GFP* and *rpr* in SE0_PG_ neurons. Those larvae that exhibited a delayed pupariation were dissected at 120 hAH and immunostained for GFP (green) and serotonin (magenta). In our experimental condition, some cells were eliminated by *rpr* but others were not. The presence of the cell bodies and the PG projections are indicated with yellow arrows and circles, while the absence of them are indicated with blue ones. In the example 1 (**f**–**h**), the right side of SE0_PG_ cell bodies and the PG projection were lost (blue arrow and circle). In the examples 2 and 3 (**i**–**n**), the PG projections remained (yellow circles) but serotonin signals significantly decreased in these PG projections (blue circles in **k**,**n**). For statistical analysis, Student’s *t*-test was performed in **b**,**c**,**e**. *P<0.05. The average values of triplicate data sets are shown with s.e.m.. Bars: (**b**) 1.0 mm; (**f**–**n**) 50 μm. Each experiment was conducted independently at least three times.

To inhibit the function of serotonergic SE0_PG_ neurons, we expressed *tetanus toxin light chain (TeTxLC)*, a neuron-specific toxin that prevents the presynaptic release of synaptic vesicles^34^, using *R29H01–GAL4* or *TRH–GAL4*. The timing of pupariation was delayed by 1–2 days in *R29H01>TeTxLC* as well as *TRH>TeTxLC* larvae (Fig. 4a and Supplementary Fig. 2b). As a result, *TeTxLC*-expressing larvae became giant in size, which is a typical phenotype of ecdysteroid deficiency (Fig. 4b)^8^. This phenotype was not observed when the inactive forms of *TeTxLC* were expressed (Supplementary Fig. 2a). Next, we measured the ecdysteroid titres in *R29H01>TeTxLC* and control larvae (Fig. 4c). In control larvae, the ecdysteroid titres increase from the late third instar stage (96 hAH) to the prepupal stage (96–120 hAH). The substantial increase of the ecdysteroid titres is associated with pupariation, a transition of development^35^. In contrast, the ecdysteroid levels were not significantly elevated in *R29H01>TeTxLC* larvae, suggesting that the ecdysteroid peak is absent in these animals. Consistent with this, the expression levels of ecdysteroidogenic genes were significantly reduced in the late third instar stage (Fig. 4e). Moreover, we found that the timing of pupariation was restored when *R29H01>TeTxLC* larvae were fed with food containing 20E (Fig. 4a). These results suggest that the phenotype for delay in the pupariation timing is due to the impairment of E biosynthesis.

To reduce the possibility that neurons other than SE0_PG_ neurons are involved in the pupariation timing, we genetically manipulated the expression of *TeTxLC* only in three pairs of SE0 neurons by using the Q system in conjunction with the GAL4 system (Fig. 4d). The pupariation timing of these larvae showed a delay, similar to that in *R29H01>TeTxLC* or *TRH>TeTxLC* larvae, although *GFP*-expressing control larvae also showed a milder delay at 96 hAH. This result strengthens the hypothesis that SE0_PG_ neurons are responsible for the proper timing of E biosynthesis at pupariation.

We also examined whether *TeTxLC* expression in SE0_PG_ neurons caused a delayed pupariation timing under the yeast-poor condition. Because SE0_PG_ neurons hardly innervate the PG cells on yeast-poor food, *TeTxLC* expression would not further affect on the delayed timing of pupariation. As we expected, control and *R29H01>TeTxLC* larvae became pupae at the similar time course of 144–216 hAH in the yeast-poor condition (Supplementary Fig. 4a). Furthermore, *TeTxLC* expression reduced, but did not eliminate, the effect of poor food on pupariation timing (Supplementary Fig. 4a,b). These results suggest that nutrient-dependent control of pupariation timing is partially, but not fully, mediated by SE0_PG_ neurons.

As an alternative approach to manipulating neuronal activities in a small group of cells, we utilized mosaic analysis with a repressible cell marker (MARCM)^36^ to express genes selectively in a subpopulation of serotonergic neurons that included SE0_PG_ neurons. We used the cell death gene *reaper* (*rpr*) to inhibit the function of SE0_PG_ neurons. After inducing mitotic clones through heat shock, a group of larvae expressing both *GFP* and *rpr* had a prolonged larval stage compared with the control. When we dissected those larvae that exhibited a delayed pupariation (at 120 hAH), some SE0_PG_ neurons were labelled with GFP (yellow arrows in Fig. 4g,j, and m), indicating that *rpr* did not always eliminate cells under our experimental condition. Other cells were not clearly labelled with GFP (blue arrows in Fig. 4g,j), possibly because they were eliminated by *rpr* or did not make mitotic clones. Nevertheless, the serotonin signal was severely decreased in all of the SE0_PG_ axons that we observed at 120 hAH (Table 1; blue circles in Fig. 4h,k and n). In contrast, when the wandering larvae expressing both *GFP* and *rpr* were dissected at 96 hAH, only 40% of the animals showed a decreased serotonin signal (Table 1). These data suggest that the reduction of serotonin in SE0_PG_ neurons strongly correlates with a prolonged larval stage, which reflects the timing of delayed E biosynthesis.

### Serotonin regulates the timing of E biosynthesis in the PG

**Figure 5:**
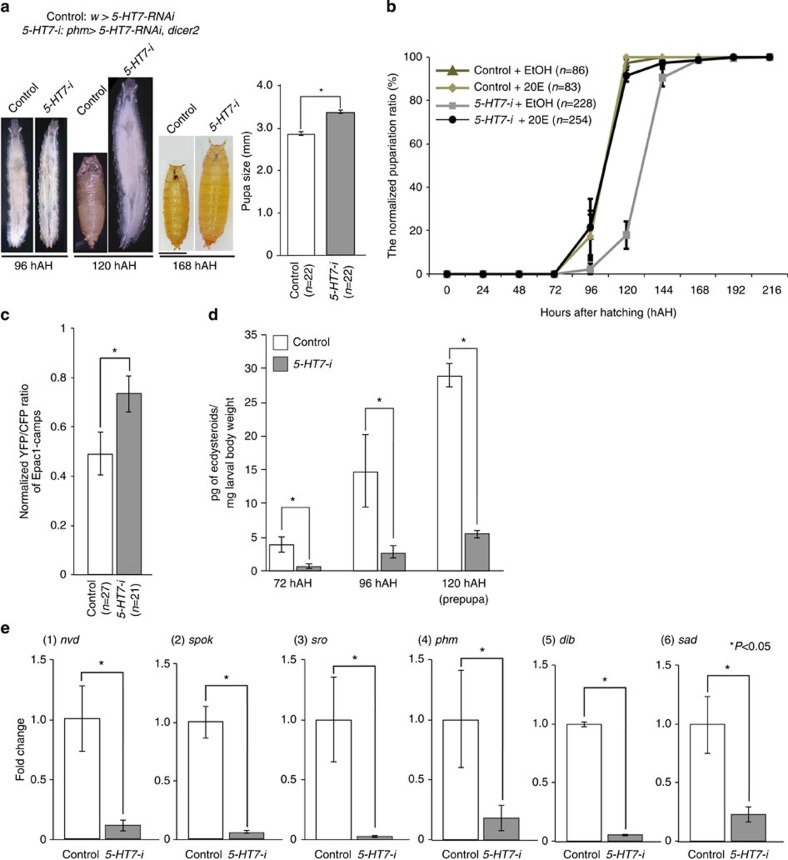
Serotonin signalling promotes ecdysteroid biosynthesis. (**a**) Representative examples of control (*w>5-HT7–RNAi*) and the PG-specific *5-HT7–RNAi* animals, indicated as *5-HT7-i* in the Figures (*phm>5-HT7–RNAi, dicer2*). Samples were collected at 96, 120 and 168 hAH. *5-HT7-i* animals gave rise to an increased size of larvae and pupae. The graph shows the average values of pupa size (mm) in control and *5-HT7–RNAi* animals (*n*=22) and standard errors (s.e.). (**b**) The pupariation ratio after hatching. When newly hatched larvae were raised at 25 °C, control larvae pupariated at 96–120 hAH. The timing of pupariation was delayed in *5-HT7-i* larvae by 1–2 days (120–144 hAH; see also Supplementary Fig. 3). This developmental delay was rescued by oral administration of 0.1 mg of 20E per gram of fly food. The number of animals: control (*n*=86, EtOH; *n*=83, 20E) and *5-HT7-i* (*n*=228, EtOH; *n*=254, 20E). (**c**) The measurement of cAMP levels with the Epac1–camps FRET probe. The PGs expressing *Epac1–camps* were collected in the third instar stage of control and *5-HT7-i* larvae. The normalized YFP/CFP ratio increased in *5-HT7-i* animals, indicating that cAMP levels decreased. The average values of >20 individual samples are shown with s.e.m. (**d**) The levels of ecdysteroids in control and *5-HT7-i* larvae at 72, 96 and 120 hAH. At 120 hAH, prepupae were collected from the control. The ecdysteroid titre did not increase in *5-HT7-i* larvae. (**e**) Quantitative reverse transcription–PCR analysis revealed that the expression of ecdysteroidogenic genes decreased in the RG of *5-HT7-i* larvae in the late third instar stage (96 hAH). The fold changes compared with control larvae (*w>5-HT7–RNAi*) were calculated. (1) *nvd*, (2) *spok*, (3) *sro*, (4) *phm*, (5) *dib*, (6) *sad*. For statistical analysis, the average values of triplicate data sets are shown with s.e.m. in **d**,**e**. One-way analysis of variance test was used for c, *P<0.01. Student’s *t*-test was used for **a**,**d**,**e**. *P<0.05. Bar, 1 mm. Each experiment was conducted independently at least three times.

Finally, we examined whether the serotonin signal was transmitted to the PG. The *D. melanogaster* genome encodes five serotonin receptors: 5-HT1A, 5-HT1B, 5-HT2A, 5-HT2B and 5-HT7 (ref. 37). Utilizing transgenic RNA interference (RNAi) lines targeting these receptor genes, we found that the PG-specific knockdown of one receptor gene, *5-HT7*, caused a developmental delay and an increased size (Fig. 5a,b; Supplementary Fig. 3; Supplementary Tables 1 and 2). *5-HT7* expression in the PG cells was confirmed by the observation that two independent *5-HT7–GAL4* transgenic lines were active in the PG (Supplementary Fig. 3a–c)^37^,^38^. The developmental delay phenotype was caused by either of the two independent RNAi lines that target different regions (Supplementary Fig. 3d–g; Supplementary Table 1), suggesting that the effect of the RNAi was specific to *5-HT7* and was not an off-target effect. It has previously been demonstrated that the forced expression of *5-HT7* raises the levels of the second messenger cyclic AMP (cAMP)^39^. To measure the levels of cAMP in the PG of control and *5-HT7–RNAi* larvae, we used a fluorescence resonance energy transfer (FRET) biosensor, Epac1-camps^40^. Briefly, a high level of the FRET ratio indicates a low level of cAMP. The FRET ratio is higher in the third instar stage of *5-HT7–*RNAi larvae than that of control larvae, indicating that cAMP levels are downregulated in the PG of *5-HT7–RNAi* larvae (Fig. 5c). The developmental delay phenotype was rescued by oral administration of 20E in food (Fig. 5b), suggesting that *5-HT7–*RNAi causes ecdysteroid deficiency. To further test this possibility, we compared the ecdysteroid titres between control and *5-HT7–*RNAi animals (Fig. 5d). As in *R29H01>TeTxLC* larvae, 5*-HT7*–RNAi animals had lower ecdysteroid levels during the third instar stage. Consistent with this phenotype, the expression levels of ecdysteroidogenic genes were significantly reduced in *5-HT7–*RNAi animals (Fig. 5e). Taken together, these results suggest that serotonin signalling plays an important role in ecdysteroid biosynthesis in the PG.

## Discussion

The timing of the developmental transition from the juvenile stage to adulthood is closely linked to nutrient conditions. In *D. melanogaster*, the larval stage is a feeding period for growth, and once the larvae gain sufficient weight for metamorphosis, they stop feeding and wander away from food. Therefore, nutrient signals activate the behavioural changes accompanied with ecdysteroid biosynthesis at pupariation, that is, the period of the juvenile-to-adult transition.

In this study, we provide a novel insight into the mechanism controlling pupariation timing in a nutrient-dependent context. Our results strongly suggest that a subset of serotonergic (SE0_PG_) neurons directly innervate the PG, regulating the timing of ecdysteroid biosynthesis. Strikingly, the projection of SE0_PG_ neurons is affected by nutrient conditions and is correlated with the timing of pupariation. Furthermore, serotonin signalling mediates ecdysteroid biosynthesis in the PG. Our results are novel and significant particularly in the following two aspects. First, as far as we know, this study is the first report showing that biogenic amines act on the PG *in vivo*, in contrast to a number of previous studies focusing on neuropeptides acting on the PG (ref. 21). Second, our study suggests that nutrient conditions affect ecdysteroid biosynthesis via the direct neuronal projections to the PG from the insect feeding centre SOG. Given that serotonin modulates neuronal activities in response to food stimuli^41^, our result sheds light on a novel role for serotonin in linking nutrient conditions to steroid hormone biosynthesis, underlying the progression of development in concert with nutrient availability.

Although it has been reported that serotonin-positive neurites innervate the CC in the RG (refs 20, 23), our careful microscopic observation has revealed that a few serotonergic neurites of SE0_PG_ neurons directly project to the PG, the ecdysteroid-producing organ (Fig. 1c). The projection pattern of SE0_PG_ neurons is distinct from that of PTTH neurons, which cover the entire region of the PG. Currently it is unclear whether serotonin signalling is transduced equally to all the PG cells. However, it should be noted that serotonergic neurotransmission can best be described as volume transmission, in which the majority of uptake sites appear to be located beyond synaptic junctions^42^. Thus, we hypothesize that serotonin is released into the extracellular space of the PG and is allowed to diffuse sufficient distances to activate serotonin receptors on all cells of the PG. Alternatively, it is also possible that only a few PG cells might receive serotonin signals that are then propagated by the secondary signals to the neighbouring PG cells. For example, Ca^2+^ influx is detected downstream of PTTH signalling in the lepidopteran species^43^,^44^,^45^. Such a mechanism would allow PG cells to synchronize the timing of ecdysteroid biosynthesis at one time.

Through anatomical studies, we have revealed that SE0_PG_ neurons belong to the stomatogastric nervous system, which controls the movements of the foregut and pharynx associated with feeding behaviours (Fig. 1g)^46^. Our GFP reconstitution across synaptic partners analysis strongly suggests that SE0_PG_ neurons have synaptic contact with *hug*-expressing SOG neurons, but this must be verified through ultrastructural analysis (Fig. 2g). Considering that the activation of *hug*-expressing neurons reduces feeding behaviour and increases wandering-like behaviours^47^, one attractive scenario is that *hug* neurons and SE0 neurons are involved in a switch of post-feeding behaviours by promoting ecdysteroid biosynthesis in the late third instar stage. Both *hug* and SE0 neurons have several distinct target regions including the RG, so there should be a functional subdivision or a specific connection between the feeding circuit and neuroendocrine system. To address this question, identification of SE0_PG_ cell bodies in the SE0 cluster will be required so that their activity can be monitored in the context of developmental transitions. Although our trial of generating single-cell clones failed to determine the exact number of SE0_PG_ neurons, alternative sets of GAL4 drivers could successfully narrow down the numbers of candidate cells (Fig. 2a–e).

The phenotype obtained after the suppression of serotonin signalling is a developmental delay and a increased body size (Fig. 5a,b), which is typical for ecdysteroid deficiency and remarkably similar to the phenotype after IIS/TOR pathway inhibition in the PG (refs 15, 16, 17, 18). It is feasible to think that serotonin signalling would cooperatively regulate the timing of ecdysteroid biosynthesis with the IIS/TOR pathway. It has been reported the elevated level of serotonin suppresses the systemic IIS pathway, resulting in a developmental delay and a reduced body size^48^. This previous result suggests that serotonin plays a negative role in promoting developmental timing. In contrast, we argue that this mechanism appears to be distinct from the function of SE0_PG_ neurons, as SE0_PG_ neurons positively regulate developmental timing via ecdysteroid biosynthesis (Fig. 4). Therefore, it is likely that another set of serotonergic neurons regulates body size and developmental timing via the systemic IIS pathway. There are more than 100 serotonergic neurons in the larval brain^23^, and the local and systemic actions of signalling affect on body size and developmental timing in different ways. Thus, the balance of serotonin signalling might contribute to the proper timing of development.

**Figure 6:**
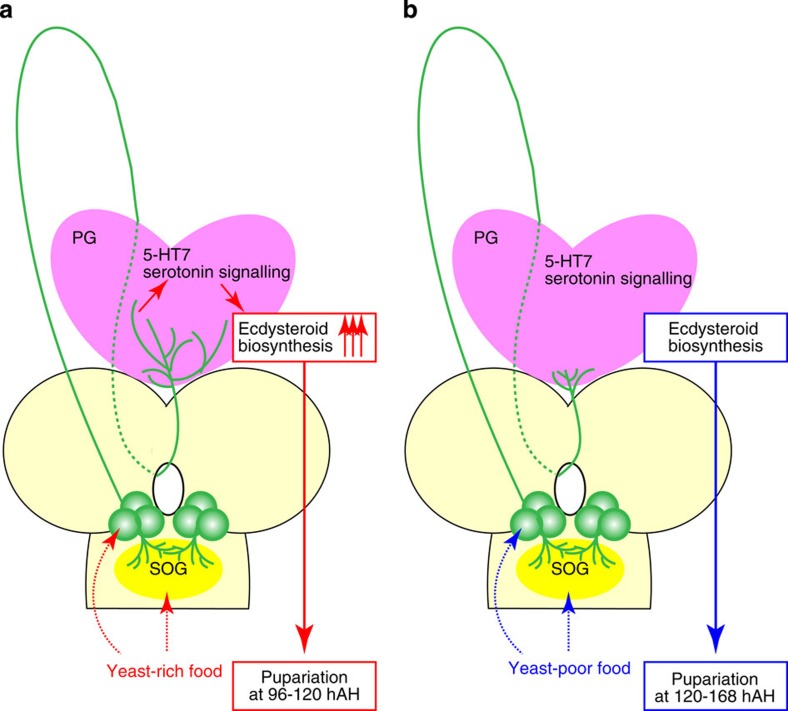
A model of serotonergic SE0_PG_ neuron signalling in response to nutrition. (**a**) On yeast-rich (or regular) food, SE0_PG_ neurons innervate the PG and activate serotonin signalling in the PG. Serotonin signalling accelerates ecdysteroid biosynthesis by upregulating the expressions of ecdysteroidogenic genes in the late third instar stage. This leads to the larva-to-pupa transition (pupariation) at 96–120 hAH. (**b**) On yeast-poor food, SE0_PG_ neurons barely project to the PG so that serotonin signalling is not activated. In this case, the timing of pupariation is delayed to 120–168 hAH.

One of the important questions that remain to be elucidated is how the neurite extension and retraction of SE0_PG_ neurons are regulated in response to nutrients at the molecular and cellular levels. Recent studies have focused on the morphological plasticity of neurons or tracheas under the variation of environmental challenges. For example, in the visual system, variation of the sensory inputs induced substantial morphological plasticity in the dendritic arbours of the postsynaptic neurons^49^. On the other hand, nutrient-dependent plasticity of the tracheal branching is directly regulated by nutrient-responsive neurons^50^. In this study, we found that the projection of SE0_PG_ neurons was affected by yeast concentration in food (Fig. 3). Yeast contains amino acids as well as ergosterol, which is the major yeast sterol that is utilized for ecdysteroid biosynthesis in *Drosophila*^51^,^52^,^53^. One possibility is that SE0_PG_ neurons directly sense the level of amino acids and/or ergosterol in haemolymph, and thus change their neurite morphology (Fig. 6). Alternatively, amino acids and/or ergosterol may be sensed by some gustatory receptor neurons (GRNs) because GRNs are known to project to SOG (ref. 54). In this case, the activated GRNs would modulate the *hug*-expressing SOG neurons and then transmit the nutrient signal to SE0_PG_ neurons. A previous study reports that the biogenic amine dopamine modulates the projection patterns of serotonergic neurons in the feeding circuit^41^, suggesting that neuronal inputs from other neurons can also induce structural plasticity in the neuroendocrine circuit. It will be interesting to examine whether the IIS/TOR pathway, a nutrient-sensing mechanism, is associated with cytoskeletal reorganizations in SE0_PG_ neurons.

Based on our observation, we propose that SE0_PG_ neurons and serotonin signalling accelerate ecdysteroid biosynthesis at pupariation in response to nutrition. In the regular or rich food condition, SE0_PG_ neurons innervate the PG and activate serotonin signalling in the PG (Fig. 6a). Serotonin signalling upregulates cAMP level, promoting ecdysteroid biosynthesis in the late third instar stage. This signalling is important for ensuring a drastic increase of ecdysteroids in the nutrient-dependent manner. Among developmental transitions in *Drosophila*, the larva-to-pupa transition, that is, ‘pupariation’, is particular sensitive for nutrient^3^, whereby serotonin signalling is activated. Once the function of SE0_PG_ neurons or serotonin receptor is disrupted, serotonin signalling does not accelerate ecdysteroid biosynthesis. The low ecdysteroids titre results in a delay of pupariation by 1–2 days. As *TeTxLC* expression or *5-HT7–RNAi* did not impair pupariation itself, serotonin signalling is essential for the timing, but not the progression of developmental transition. It is likely that other signalling pathways including PTTH pathway, IIS/TOR pathway or TGF-β pathway compensate serotonin signalling. On the other hand, in the yeast-poor condition, SE0_PG_ neurons barely project to the PG so that serotonin signalling is not activated (Fig. 6b). As a result, the timing of ecdysteroid biosynthesis is delayed. It is worth reminding that the delayed pupariation timing in the yeast-poor condition was partially suppressed by inhibiting SE0_PG_ neuronal activity (Supplementary Fig. 4). This result strengthens our hypothesis that SE0_PG_ neurons mediate nutrient signal for the timing of ecdysteroid biosynthesis. However, dietary restriction still retards developmental timing with disrupted SE0_PG_ neurons, implying that SE0_PG_ neurons are not the only mechanism that links nutrition to developmental timing. Larvae on poor food are able to pupariate independently of signalling through SE0_PG_ neurons, so that other nutrient-sensitive mechanisms could regulate ecdysteroid biosynthesis.

The biogenic amines are ancient, evolutionarily conserved molecules that function in many physiological contexts in both vertebrates and invertebrates. In light of our *Drosophila* findings, it will be interesting to explore the serotonergic neuronal control of steroid hormone biosynthesis in other species. In the silkworm *Bombyx mori*, the PGs are also innervated by several nerves starting from the SOG (ref. 21). Although it remains to be determined whether any of the *B. mori* SOG neurons are serotonergic, these data raise the possibility of a common feeding neural circuit mechanism affecting E biosynthesis in insects. In mammals, serotonergic neurons are thought to be involved in brain glucose sensing, satiety response or cessation of feeding after food intake^55^,^56^. Taken together, serotonin signalling may constitute a link between the external nutrient conditions and the internal endocrine systems, affecting developmental plasticity by modulating the timing of developmental transition.

## Methods

### Fly strains and culture

*Drosophila melanogaster* flies were raised on standard agar–cornmeal medium at 25 °C under a 12:12 h light/dark cycle. *w*^*1118*^ was used as the wild type. Heterozygous controls were obtained by crossing GAL4 driver or UAS effector to *w*^*1118*^. The following transgenic and mutant flies were used: *phm–GAL4#22, ptth–HA* (gifts from M.B. O’Connor, University of Minnesota)^8^,^10^, *TRH–GAL4* (a gift from O. Alekseyenko, Harvard University)^25^, *5-HT7*^*Gal4*^ (a gift from R. Yao, Peking University School of Life Sciences)^37^, *5-HT*_*7*_*Dro–GAL4* (a gift from C.D. Nichols, Louisiana State University Health Sciences Centre)^38^, *hugS3–GAL4* (a gift from M. Pankratz, University of Bonn)^30^*, LexO–spGFP11::CD4; UAS–spGFP1-10::Nrx* (a gift from N. Shah, University of California, San Francisco)^33^*, UAS–turboRFP/TM6b* (a gift from A. Koto and M. Miura, The University of Tokyo), *UAS–ECFP* (a gift from T. Uemura, Kyoto University), *UAS–TrpA1; UAS–TrpA1/TM*3 (a gift from T. Kiya, Kanazawa University), *UAS–rpr* (a gift from T. Igaki, Kyoto University)^57^ and *UAS–GFP; UAS–mCD8::GFP* and *UAS–DsRed; UAS–nSyb::GFP* (gifts from K. Ito, The University of Tokyo)^58^. *R29H01–GAL4* (#47343), *UAS–dicer2* (#24650)*, tubP–GAL4* (#5138), *UAS–5-HT7–RNAi*^*JF02576*^ (#27273), *UAS–Epac–camps* (#25408), *UAS–TeTxLC* (#28837, #28838), *UAS–TeTxLC(inactive)* (#28839, #28840, #28841), *UAS–(FRTstop)–TeTxLC* (#28842), *UAS–(FRTstop)–mCD8::GFP* (#30125), *QUAS–DSCP–FLPo.2G* (#30008), *trh–QF* (#52251) and *trh–lexA::p65* (#52248) were obtained from the Bloomington *Drosophila* Stock Centre (Indiana University, Bloomington, IN, USA). *yw, hs–flp;FRTG13UAS–mCD8::GFP* (#108062) and *w; FRTG13 tubP–GAL80* (#108073) were obtained from the Kyoto *Drosophila* Genetic Resource Centre (Kyoto Institute of Technology, Kyoto, Japan). *UAS–5-HT7–RNAi*^*KK10804*^ was obtained from the Vienna *Drosophila* RNAi Centre (Vienna, Austria). The target region is in the 3′ untranslated region, which is distinct from that of *UAS–5-HT7–RNAi*^*JF02576*^.

Animals shown in figures are the following genotypes:

(Fig. 1a,c) *w; phm–GAL4/UAS–RFP*

(Fig. 1d,e) *TRH–GAL4/UAS–DsRed; TRH–GAL4/UAS–nSyb::GFP*

(Fig. 1f–i) *w; TRH–GAL4/UAS–GFP; TRH–GAL4/UAS–mCD8::GFP*

(Fig. 2a–c) *w; UAS–GFP/+; R29H01–GAL4/UAS–mCD8::GFP*

(Fig. 2d,e) w; *trh–QF/UAS–(FRT–stop–FRT)–mCD8::GFP;R29H01–GAL4/QUAS–Flp*

(Fig. 2f,g) *TRH–lexA/lexAop–spGFP11::CD4; hugS3–GAL4/UAS–spGFP1-10::Nrx.*

(Fig. 2h,i) *TRH–lexA/+; hugS3–GAL4/+*

(Fig. 3a,b) *w*^*1118*^

(Fig. 3c–h) *yw; ptth–HA-50*

(Fig. 3i–n) *TRH–GAL4/UAS–GFP; TRH–GAL4/UAS–mCD8::GFP*

(Fig. 4a–c and e) *R29H01–GAL4/UAS–TeTxLC, +/UAS–TeTxLC*

(Fig. 4d) *TRH–QF/UAS–(FRTstop)–TeTxLC; R29H01–GAL4/QUAS–Flp, TRH–QF/UAS–(FRTstop)–mCD8::GFP; R29H01–GAL4/QUAS–Flp*

(Fig. 4f–o) *yw, hs–flp/UAS–rpr; FRTG13 UAS–mCD8::GFP/FRTG13 tubP–GAL80; TRH–GAL4/+*

(Fig. 5a,b,d and e) *w; UAS–5-HT7–RNAi*^*KK10804*^*/UAS–dicer2; phm–GAL4/+*, *UAS–5-HT7–RNAi*^*KK10804*^*/+*

(Fig. 5c) *w; UAS–5-HT7–RNAi*^*KK10804*^*/UAS–dicer2; phm–GAL4/UAS–Epac1–camps*,

w; +/UAS–dicer2; phm–GAL4/UAS*–*Epac1*–*camps.

### Nutrient condition assay

Our standard fly food contains 0.5 g agar, 5.0 g glucose, 4.5 g of cornmeal, 2.0 g yeast extract and 150 μl propionic acid in 50 ml water. To change the nutrient conditions, we increased or decreased the amount of yeast extract: 5.0 g (2.5 × 2.0 g) for the yeast-rich condition and 0.4 g (0.2 × 2.0 g) for the yeast-poor condition. No yeast paste was added in the fly tubes. Approximately 22 h after egg laying, newly hatched larvae were raised on food with various amounts of yeast.

### Developmental timing analysis

Embryos were collected on grape-juice agar plates at 25 °C. Newly hatched larvae were transferred to small vials and raised on ground fly food. At the appropriate time point (hAH), staged larvae were dissected for fixation or mounted for live imaging. To determine the duration of the third instar larval stage in hours, we collected L2 larvae at 48 hAH and allowed them to be moulted in 2–6 h intervals. L3 larvae were collected within 6 h after third instar moulting (0–6 hA3L).

### Measurement of neurite projections to the PG

To quantify the projection of neurons to the PG, we used ImageJ to measure the lengths of individual neurites extending on the PG area. The PG area was defined by immunostaining of Shroud (Sro). The total length of neurites was calculated both for SE0_PG_ neurons and for PTTH neurons. To avoid effects of mounting on the slide, we carefully chose samples of the Brain–RG complex that were in a proper positional relation. We also dissected samples in a fillet to maintain the relative positions of the Brain and RG. In the data collection, we took series of confocal images to trace the entire neurite projection patterns and these confocal images were flattened along the *z*-axis to single planes. The neurite length along the *z*-axis was not considered.

### Antibodies against ecdysteroid biosynthesis enzymes

Antibodies against Sro (ref. 59) was raised in guinea pig. A synthetic peptide (NH_2_–LTVRFCAMPTYESTNRQEKI–COOH) corresponding to the C-terminal residues (316–335) of Sro amino acid sequence (GenBank accession number AB361435) were used for immunization.

### Immunohistochemistry

Larvae were dissected at the appropriate developmental stage and fixed with 3.7% formaldehyde with 0.05% Triton X-100 for 20 min at room temperature. The following primary antibodies were used: anti-serotonin (rabbit, 1:500, Sigma), anti-Sro (guinea pig, 1:1,000), anti-Pheromone biosynthesis activating neuropeptide (PBAN; rabbit, 1:200; a gift from K. Shiomi, Shinshu University)^60^ and anti–GFP (rabbit, 1:500, Molecular Probes; mouse, 1:100, Wako). The secondary antibodies used were Alexa Fluor 488/555/633 (1:200, Molecular Probes). Samples were visualized on a LSM 700 confocal microscope (Carl Zeiss). Images were processed using Adobe Photoshop CS4 or Image J version 1.43 m (ref. 61).

### Feeding experiments with 20E

The 20E was purchased from ENZO Life Sciences and Sigma. Newly hatched larvae were transferred to small vials and raised on standard fly food or semi-defined medium (FlyBase) with 0.3 mg ml^−1^ 20E or 2% ethanol (vehicle only as a control). The developmental stages were scored twice daily.

### Ecdysteroid titre measurements

Ecdysteroid titres were quantified by ELISA essentially^62^. 20E (Sigma) and 20E-acetylcholinesterase (Cayman Chemicals) were used as the standard and enzymatic tracers, respectively. Absorbance was read at 415 nm using a microplate reader Model 680 (Bio-Rad). The ecdysteroid antiserum has the same affinity for E and 20E (ref. 63), but because the standard curve was obtained with the latter compound, the results are expressed as 20E equivalents. For sample preparation, 10–30 staged larvae were weighed and homogenized in 100 μl of methanol three times. After centrifugation at 14,000 r.p.m. for 5 min, supernatants were transferred to new tubes and dried with centrifugal evaporator. Samples were resuspended in 50 μl of EIA buffer (0.1M phosphate solution containing 0.1% BSA, 0.4 M NaCl, 1 mM EDTA and 0.01% NaN_3_) and incubated at 4 °C overnight.

### Quantitative reverse transcription–PCR

Total RNA was extracted from the RGs using NucleoSpin RNA II (Takara Bio Inc.). RNA was reverse transcribed using ReverTra Ace qPCR RT Master Mix with gDNA remover (TOYOBO) and the generated complementary DNA was used as a template for quantitative PCR (qPCR) using ThunderBird SYBR qPCR mix (TOYOBO) on a Thermal Cycler Dice Real Time System (Takara Bio Inc.). The amount of target RNA was normalized to an endogenous control *ribosomal protein 49 (rp49)*, and the relative fold change was calculated. The primer sets used are shown in Supplementary Table 3. The primers for quantifying *nvd*, *spok, sro, phm*, *dib* and *sad* were used in the previous studies^8^,^59^.

### MARCM analysis

For generating mitotic clones in SE0_PG_ neurons, embryos were collected for 2 h of egg laying and incubated at 25 °C for 6 h. Then 6–8 h after egg laying, embryos were heat-shocked at 38 °C for 1 h. We tried to generate a single-cell clone expressing *GFP*. However, we could not find a heat-shock condition to induce mitotic clones at a single-cell level.

### FRET imaging

For live imaging, larvae were anesthetized with ether and placed on the glass slide with 100% glycerol. Images were obtained with a × 40 objective lens (oil-immersion) using a Zeiss LSM 700 confocal microscope. Cyan Fluorescent Protein (CFP) was excited with a 405 nm laser and its emission was detected with a band-pass filter of 465–505 nm. Yellow Fluorescent Protein–FRET (YFP–FRET) emission was detected with a long-pass filter of 525 nm. The efficiency of FRET was estimated by acceptor bleach (~20%). The ratio of YFP/CFP emissions was determined after subtracting CFP spillover into the YFP channel from the YFP intensity^40^,^64^. To determine the CFP spillover in our imaging system, we used larvae expressing only *ECFP* and obtained images through CFP/YFP-emission channels. Furthermore, the YFP/CFP ratio values were normalized to the values of the second instar stage, for which there was no statistically significant difference between the values of the control and *5-HT7–RNAi* animals. The values in each stage were compared by a single-factor analysis of variance test between control and RNAi animals.

### Statistical analysis

Statistical analysis was performed with Excel (Microsoft) and an add-in software for statistics (Excel Toukei 2011; Social Survey Research Information). The mean values were calculated with standard errors. For analyzing the values of neurite length, qPCR and ecdysteroid titres, Student’s *t*-test was applied. For FRET imaging analysis, one-way analysis of variance was applied.

## Author contributions

Y.S.-N. and R.N. designed the experiments, analyzed the data, contributed reagents and materials and wrote the manuscript. Y.S.-N. performed the experiments.

## Additional information

**How to cite this article**: Shimada-Niwa, Y. and Niwa, R. Serotonergic neurons respond to nutrients and regulate the timing of steroid hormone biosynthesis in *Drosophila*. *Nat. Commun.* 5:5778 doi: 10.1038/ncomms6778 (2014).

## Supplementary Material

Supplementary Information Supplementary Figures 1-4 and Supplementary Tables 1-3

## Figures and Tables

**Table 1:** The percentage of larvae that showed lower serotonin signals in SE0_PG_ neurons.

**GFP**	**Serotonin**	**96 hAH**	**120 hAH**
**−**	**−**	30 (*n*=9)	17.9 (*n*=5)
**+**	**−**	10 (*n*=3)	82.1 (*n*=23)
**+**	+	60 (*n*=18)	0 (*n*=0)
	%	100	100

GFP, green fluorescent protein; hAH, hours after hatching; MARCM, mosaic analysis with a repressible cell marker; PG, prothoracic gland; *rpr*, *reaper*.

The ‘GFP[–] and serotonin[−]’ group indicates that SE0_PG_ neurons were lost by *rpr*. The ‘GFP[+] and serotonin[−]’ group indicates that SE0_PG_ neurons were present but the serotonin signal decreased. The ‘GFP[+] and serotonin[+]’ group indicates that SE0_PG_ neurons and the serotonin signal were detected on the PG. When the MARCM clone-induced larvae were dissected at 96 hAH (the wandering stage), 40% of these animals showed decreased level of serotonin in SE0_PG_ neurons. In contrast, when the larvae delayed for pupariation were dissected at 120 hAH, all animals decreased serotonin signals in SE0_PG_ neurons. Thus, the inhibition of SE0_PG_ neurons leads to decreased level of serotonin, which correlates with the delayed timing of pupariation.
