# Supplementary material for: Serotonergic neurons respond to nutrients and regulate the timing of steroid hormone biosynthesis in Drosophila
Source: Nat Commun. 2014 Dec 15;5:5778. doi: 10.1038/ncomms6778 (PMC4284655; doi:10.1038/ncomms6778)
Supplement: Supplementary Information — Supplementary Figures 1-4 and Supplementary Tables 1-3 [file ncomms6778-s1.pdf]

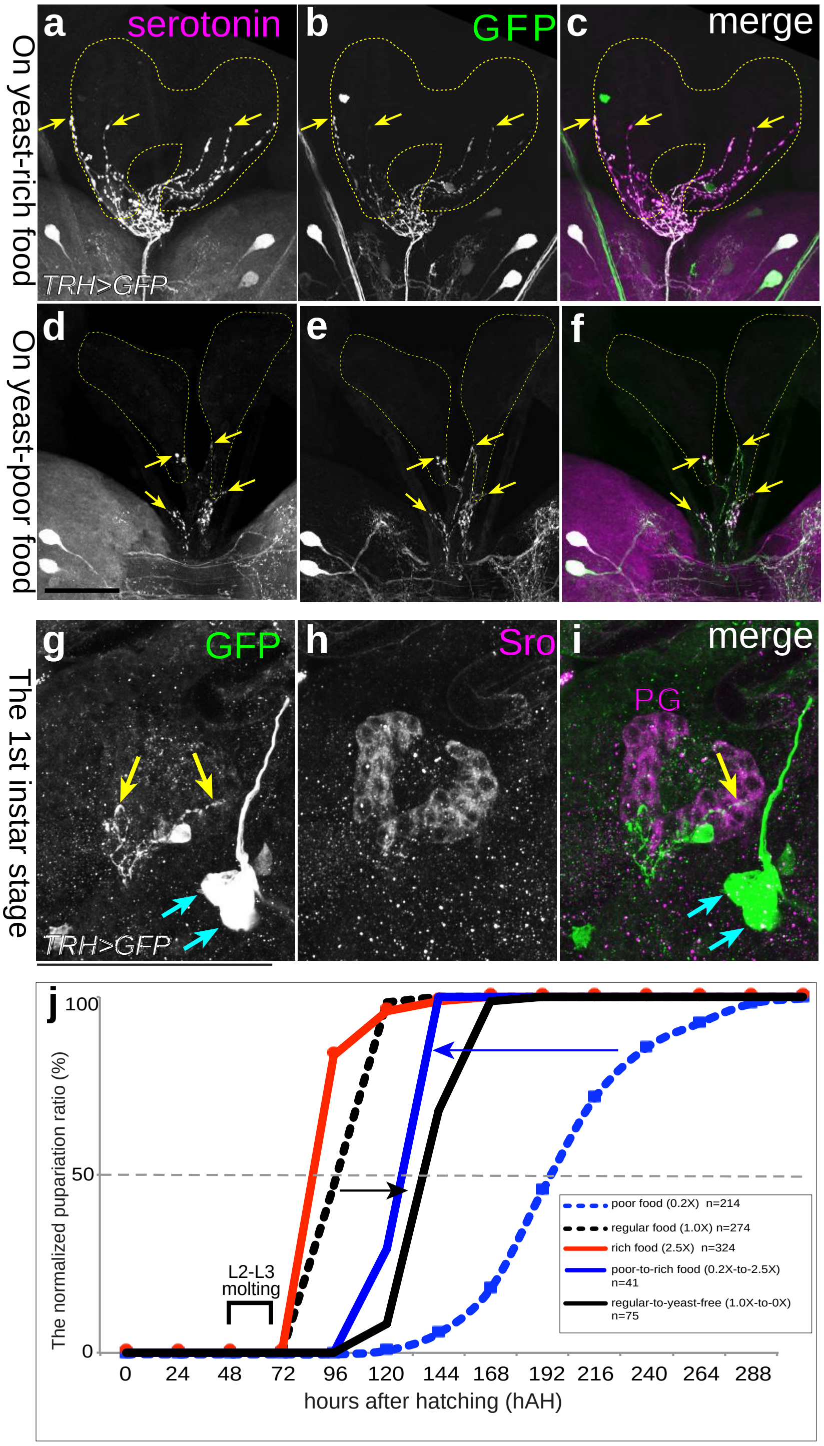

Supplementary Figure 1: The projection patterns of $SE0_{PG}$ neurons were affected by food conditions

(a-f) The Br-RG complexes of the late 3rd instar stage of *TRH>GFP* larvae raised on the yeast-rich (2.5✕ yeast, a-c) or yeast-poor (0.2✕ yeast, d-f) food conditions. Samples were

immunostained for serotonin (magenta) and GFP (green). The serotonin signals were localised at the tips of $SE0_{PG}$ neurons (arrows), indicating that serotonin is transported to the axon termini in both conditions. Both the distribution of the neurotransmitters and the projection pattern were affected by food conditions. **(g-i)** *TRH>GFP* embryos were placed on a 3.0% agar plate and left to hatch. Newly hatched larvae were picked up and dissected. Serotonergic neurons were labelled with GFP **(g)** and the PG was labelled with Sro **(h)**. $SE0_{PG}$ neurons innervated the PG at the 1st instar stage before food uptake (yellow arrows). The blue arrows indicate the cell bodies of SE0 neurons in the brain behind the PG. **(j)** The normalised pupariation ratio in response to food condition changes at the 3rd instar stage. When the 1st instar larvae were raised on the yeast-poor food (0.2× yeast) and transferred to the yeast-rich food (2.5× yeast) in the early 3rd instar stage (~6 h after L2-L3 moulting, A3L), the timing of pupariation recovered by 3 days (blue lines). Conversely, when larvae were transferred from regular food (1× yeast) to yeast-free food (0× yeast), the timing of pupariation was delayed by 1.5 days (black lines). These results suggest that the timing of pupariation responds to nutrient conditions after the 3rd instar stage. The genotypes shown is *TRH-GAL4/UAS-GFP; TRH-GAL4/UAS-mCD8::GFP*. Bar, 50 μm. Each experiment was conducted independently 3 times.

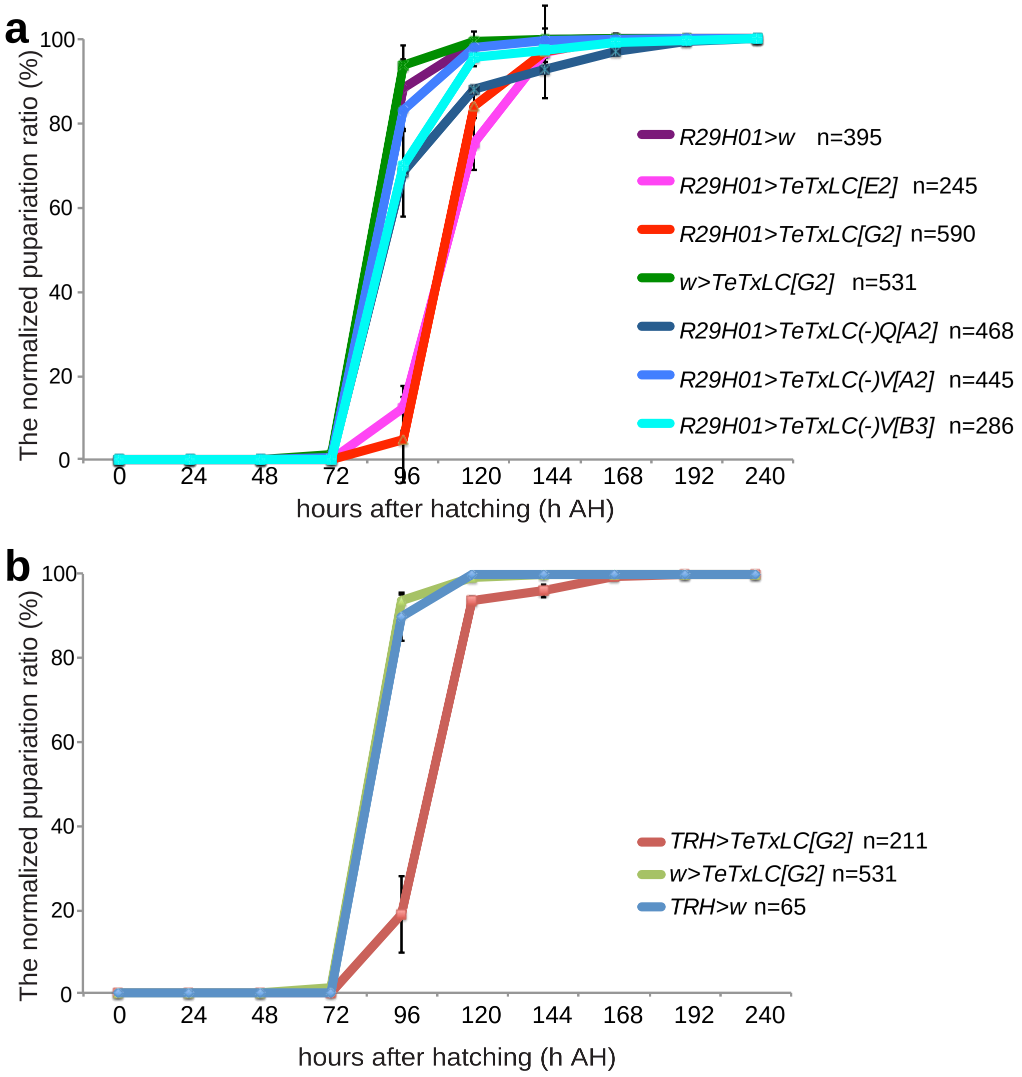

Supplementary Figure 2: Expression of *TeTxLC* with *R29H01-GAL4* or *TRH-GAL4* delayed the timing of pupariation.

Embryos were collected at 25°C, and newly hatched larvae were raised at 29°C. Most of the control larvae became pupae at 72-96 hours after hatching (hAH), whereas *R29H01>TeTxLC* **(a)** or *TRH>TeTxLC* **(b)** larvae delayed the timing of pupariation to 96-144 hAH. The expression of the inactive forms of *TeTxLC (TNT-IMP-Q* or *TNT-IMPV)* did not delay the timing, suggesting that the inhibition of *R29H01-GAL4*-positive or *TRH-GAL4*-positive

neurons is involved in the timing of pupariation. The percentages were normalised to the final number of living pupae for each genotype: (a) *R29H01-GAL4/+* (n=395), *UAS-TeTxLC[E2]/+; R29H01-GAL4/+* (n=245), *UAS-TeTxLC[G2]/+; R29H01-GAL4/+* (n=590), *UAS-TeTxLC[G2]/+* (n=531), *UAS-TeTxLC.(-)Q[A2]/+; R29H01-GAL4/+* (n=468), *UAS-TeTxLC.(-)V[A2]/+; R29H01-GAL4/+* (n=445), *UAS-TeTxLC.(-)V[B3]/R29H01-GAL4* (n=286), (b) *TRH-GAL4/UAS-TeTxLC[G2]; TRH-GAL4/+* (n=211), *TRH-GAL4/+; TRH-GAL4/+* (n=65). Each experiment was conducted independently 4 times and error bars represent standard error of the mean (SEM).

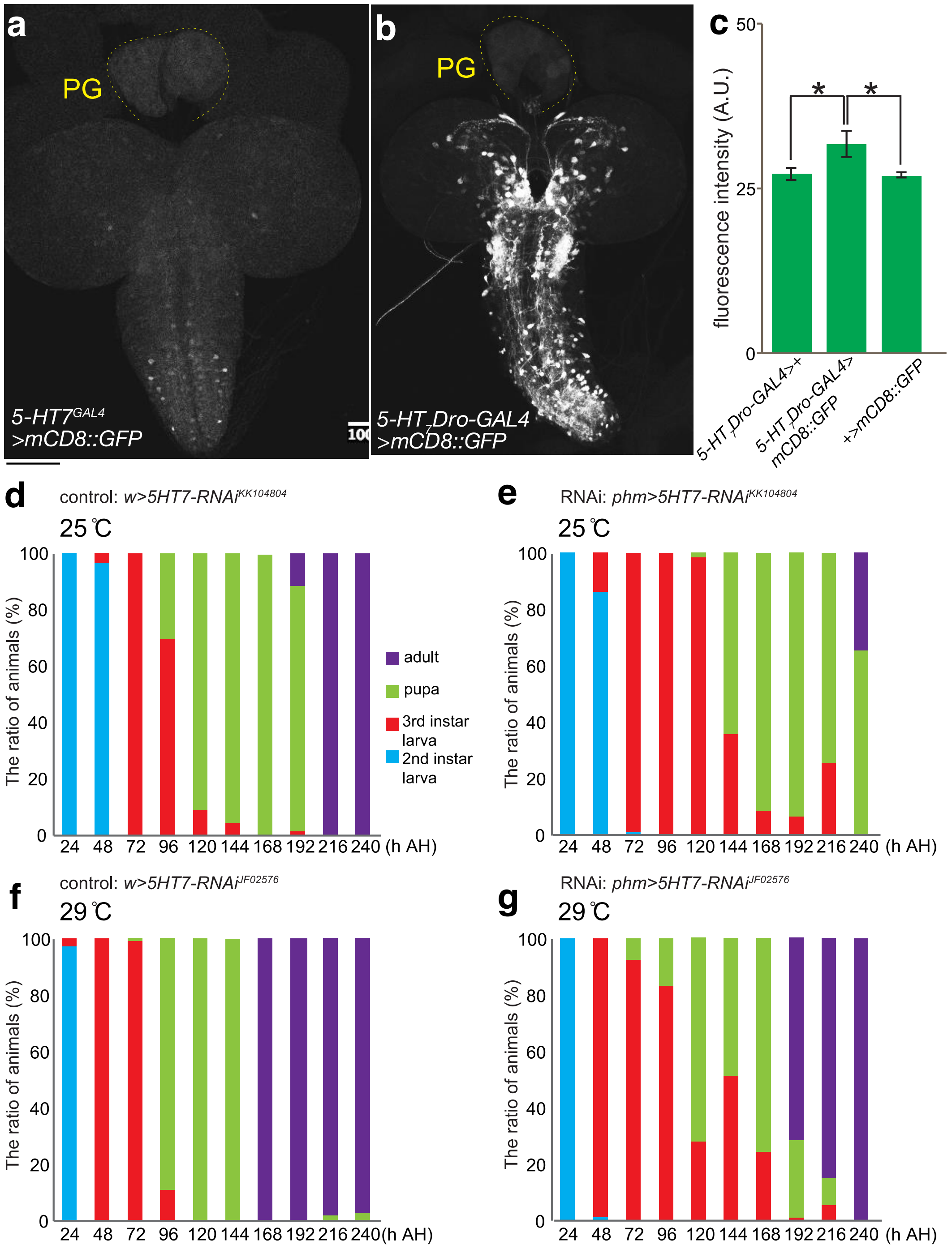

Supplementary Figure 3: The expression of *5-HT7* in the PG and the developmental profiles of PG-specific *5-HT7-RNAi* animals.

(a and b) *GFP* expression driven by *5HT7*^{Gal4} or *5-HT$_7$Dro-GAL4* was detected in the PG (outlined by dotted lines). **(c)** The quantitative analysis of GFP fluorescence in the PG of *5-HT$_7$Dro-GAL4>+* larvae (control), *5-HT$_7$Dro-GAL4>UAS-mCD8::GFP* larvae, *+>UAS-mCD8::GFP* larvae (control). The average values of >10 individual samples are shown with SEM. Student's *t*-test, *p<0.05. **(d-g)** The developmental profiles of 2 independent RNAi lines (KK10804 from VDRC and JF02576 from TRiP) were examined. These RNAi lines target distinct regions of the *5-HT7* mRNA, ensuring that the knockdown was gene-specific. The *x*-axis indicates hours after hatching. The *y*-axis indicates the ratio of animals at each stage (2nd instar larva, blue; 3rd instar larva, red; pupa, green; and adult, purple). Compared to control larvae (*w>5-HT7-RNAi*^{KK10804} in d and *w>5-HT7-RNAi*^{JF02576} in f), *5-HT7-i* larvae (*phm>dicer2, 5-HT7-RNAi*^{KK10804} in e and *phm>dicer2, 5-HT7-RNAi*^{JF02576} in g) exhibited developmental delay for 2 days at 25°C and 29°C, respectively. Each experiment was conducted independently at least 3 times.

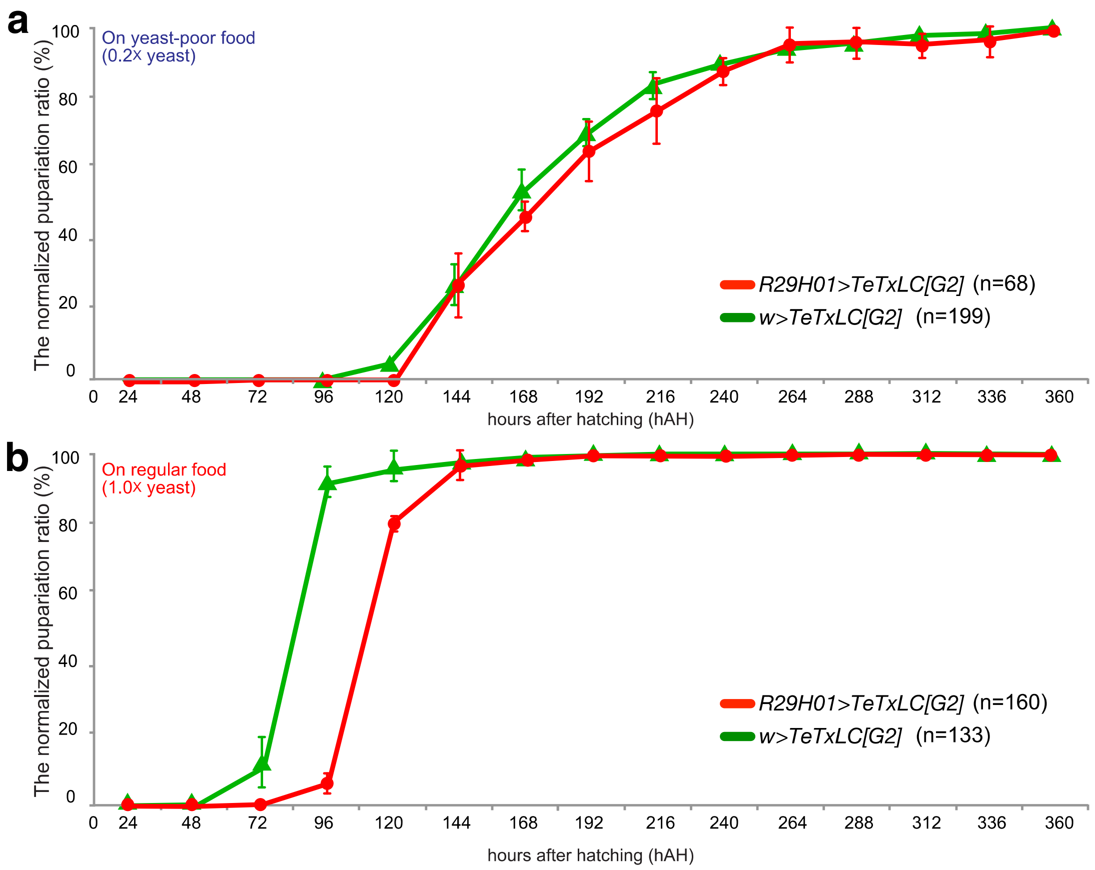

Supplementary Figure 4: A delay in the pupariation timing in *R29H01>TeTxLC* larvae was not further retarded in the yeast-poor condition

The pupariation ratios after hatching were shown. **(a)** When newly hatched larvae were raised at 29°C in the yeast-poor condition (0.2✕ yeast), control and *R29H01>TeTxLC* larvae became pupae at the similar time course of 144-216 hAH. **(b)** In contrast, *TeTxLC*-expressing larvae delayed the timing of pupariation by 1-2 days on the regular food condition (1✕ yeast). The same result was obtained in Fig. 4a. Note that the difference in the red lines between (a) and (b) is substantial but smaller than the difference in the green lines between (a) and (b). These data suggest that nutrient-dependent control of pupariation timing is partially, but not fully, mediated by $SE0_{PG}$ neurons. The percentages were normalised to the final number of

living pupae for each genotype: *UAS-TeTxLC[G2]/+; R29H01-GAL4/+* (n=68 in a, 160 in b), *UAS-TeTxLC[G2]/+* (n=199 in a, 133 in b). Fly food containing 0.2✕ or 1✕ yeast was cooked in the same day and the experiments (a and b) were performed side by side. Each experiment was conducted independently 4-5 times and error bars represent SEM.

Supplementary Table 1: The effects of RNAi for serotonin receptor genes in the PG

The PG-specific RNAi was tested against 5 serotonin receptors. In all crosses, RNAi larvae were raised at 29°C to increase *GAL4* expression levels. "+" means that the animals showed no obvious phenotypes and purariated during 96-120 hours after hatching (hAH), while "-" means that the animals did not pupariate at 96-120 hAH. n.d., not determined. Only *5-HT7-RNAi* larvae exhibited lethality or developmental delay. The phenotype was rescued by feeding with 20E. Each experiment was conducted independently 2 times.

gene name	CG number	UAS-RNAi lines		GAL4 lines		20E feeding rescue exp.
		VDRC	TRiP	X *phm-GAL4*	X *2-286-GAL4*	
5-HT1A	CG16720	KK106094		+	+	n.d.
			JF10852	+	+	n.d.
5-HT1B	CG15113	GD9558		+	+	n.d.
		GD46485		+	+	n.d.
5-HT2A	CG1056	KK102105		+	+	n.d.
5-HT2B	CG42796	GD51385		+	+	n.d.
		GD51427		+	+	n.d.
		KK102356		+	+	n.d.
5-HT7	CG12073	KK104804		-	-	rescued
			JF02576	-	n.d.	rescued

Supplementary Table 2: The effect of *5-HT7* RNAi with different GAL4 driver

Several different GAL4 drivers were tested for the tissue-specificity of *5-HT7-RNAi*. When crossed with *phm-GAL4, 2-286-GAL4, daughterless-GAL4, and tubulin-GAL4, 5-HT7-RNAi* larvae exhibited lethality or developmental delay (coloured). Other tested GAL4s produced no visible phenotype. "“+” means that the animals showed no obvious phenotypes and purariated during 96-120 hours after hatching (hAH), while “-“ means that the animals did not pupariate at 96-120 hAH. Each experiment was conducted independently 2 times.

GAL4 lines	tissues	*UAS-5-HT7-RNAi[KK104804]*
phm-GAL4	PG	**-**
2-286-GAL4	PG, epithelium	**-**
daughterless-GAL4	whole body	**-**
tubulin-GAL4	whole body	**-**
elav-GAL4	nervous system	+
breathless-GAL4	trachea	+
Lsp-GAL4	fat body	+
yp1-GAL4	fat body	+
engrailed-GAL4	posterior compartment	+
sca-GAL	wing	+

Supplementary Table 3. Primer sets used for qPCR

gene name	forward primer sequence (5'–3')	reverse primer sequence (5'–3')
rp49	ACAAATGGCGCAAGCCCAAGG	ATGTGGCGGGTGCGCTTGTT
nvd	GGAAGCGTTGCTGACGACTGTG	TAAAGCCGTCCACTTCCTGCGA
spok	TATCTCTTGGGCACACTCGCTG	GCCGAGCTAAATTTCTCCGCTT
sro	CCACAACATCAAGTCGGAAGGAGC	ACCAGGCGAATGGAATCGGG
phm	GGATTTCTTTCGGCGCGATGTG	TGCCTCAGTATCGAAAAGCCGT
dib	TGCCCTCAATCCCTATCTGGTC	ACAGGGTCTTCACACCCATCTC
sad	CCGCATTCAGCAGTCAGTGG	ACCTGCCGTGTACAAGGAGAG
